# Patient-reported outcomes of upadacitinib versus abatacept in patients with rheumatoid arthritis and an inadequate response to biologic disease-modifying antirheumatic drugs: 12- and 24-week results of a phase 3 trial

**DOI:** 10.1186/s13075-022-02813-x

**Published:** 2022-06-24

**Authors:** Martin Bergman, Namita Tundia, Naomi Martin, Jessica L. Suboticki, Jayeshkumar Patel, Debbie Goldschmidt, Yan Song, Grace C. Wright

**Affiliations:** 1 Drexel University College of Medicine, Philadelphia, PA USA; 2 AbbVie Inc., North Chicago, IL USA; 3 Analysis Group, Inc., Boston, MA USA; 4 Grace C Wright MD PC; Association of Women in Rheumatology; United Rheumatology, New York, NY USA

**Keywords:** Rheumatoid arthritis, Patient-reported outcomes, Upadacitinib, JAK inhibitors, Targeted synthetic DMARDs

## Abstract

**Background:**

In previous clinical trials, patients with active rheumatoid arthritis (RA) treated with upadacitinib (UPA) have improved patient-reported outcomes (PROs). This post hoc analysis of SELECT-CHOICE, a phase 3 clinical trial, evaluated the impact of UPA vs abatacept (ABA) with background conventional synthetic disease-modifying antirheumatic drugs (csDMARDs) on PROs in patients with RA with inadequate response or intolerance to biologic disease-modifying antirheumatic drugs (bDMARD-IR).

**Methods:**

Patients in SELECT-CHOICE received UPA (oral 15 mg/day) or ABA (intravenous). PROs evaluated included Patient Global Assessment of Disease Activity (PtGA) by visual analog scale (VAS), patient’s assessment of pain by VAS, Health Assessment Questionnaire Disability Index (HAQ-DI), morning stiffness duration and severity, 36-Item Short Form Health Survey (SF-36), Functional Assessment of Chronic Illness Therapy-Fatigue (FACIT-F), Work Productivity and Activity Impairment (WPAI), and EQ-5D 5-Level (EQ-5D-5L) index score. Least squares mean (LSM) changes from baseline to weeks 12 and 24 were based on an analysis of covariance model. Proportions of patients reporting improvements ≥ minimal clinically important differences (MCID) were compared using chi-square tests.

**Results:**

Data from 612 patients were analyzed (UPA, *n*=303; ABA, *n*=309). Mean age was 56 years and mean disease duration was 12 years. One-third received ≥2 prior bDMARDs and 72% received concomitant methotrexate at baseline. At week 12, UPA- vs ABA-treated patients had significantly greater improvements in PtGA, pain, HAQ-DI, morning stiffness severity, EQ-5D-5L, 2/4 WPAI domains, and 3/8 SF-36 domains and Physical Component Summary (PCS) scores (*P*<0.05); significant differences persisted at week 24 for HAQ-DI, morning stiffness severity, SF-36 PCS and bodily pain domain, and WPAI activity impairment domain. At week 12, significantly more UPA- vs ABA-treated patients reported improvements ≥MCID in HAQ-DI (74% vs 64%) and SF-36 PCS (79% vs 66%) and 4/8 domain scores (*P*<0.05).

**Conclusions:**

At week 12, UPA vs ABA treatment elicited greater improvements in key domains of physical functioning, pain, and general health and earlier improvements in HAQ-DI. Overall, more UPA- vs ABA-treated patients achieved ≥MCID in most PROs at all timepoints; however, not all differences were statistically significant. These data, however, highlight the faster response to UPA treatment.

**Trial registration:**

NCT03086343, March 22, 2017.

**Supplementary Information:**

The online version contains supplementary material available at 10.1186/s13075-022-02813-x.

## Introduction

Patients with rheumatoid arthritis (RA) frequently experience pain, fatigue, and impaired physical functioning that may impact their health-related quality of life (HRQOL) and ability to work and participate in daily activities [1–4]. Relief of pain is an important treatment outcome for patients and a primary reason for seeking medical care [2]. The restrictions on patients’ daily work and social activities due to symptom burden have a significant impact on their financial and social well-being [2, 4, 5]. With a significant burden on HRQOL, treatment decisions are recommended as a shared decision between the patient and physician [6]. Guidelines recommend conventional synthetic disease-modifying antirheumatic drugs (csDMARDs), such as methotrexate (MTX), as a first-line treatment strategy and biologic DMARDs (bDMARDs) like abatacept (ABA), anti-TNF inhibitors, or Janus kinase (JAK) inhibitors as second-line therapy options [6]. Unfortunately, up to 43% of patients do not respond to first-line csDMARD therapy and as many as two-thirds who receive bDMARDs have an inadequate response (bDMARD-IR) after 1 year of therapy [7, 8]. Thus, this population is difficult to adequately treat and, as such, exhibits marked increases in healthcare resource utilization; bDMARD-IR patients experienced up to 7-fold increases in hospital length of stay, admissions, and emergency department visits as compared with patients that responded to bDMARD therapy [7].

Discordance between healthcare provider and patient perceptions of disease exists [9, 10], especially in patients continuing to experience pain despite inflammation being controlled [11] or by those continuing to experience fatigue despite achieving remission [12]. To fully understand the disease burden and benefits of treatment from the perspective of patients with RA, it is important to include PROs as part of clinical trials and evaluation of treatment efficacy. This is especially true for patients with inadequate response to csDMARDs (csDMARD-IR) and bDMARD-IR. Treatment with upadacitinib (UPA), an oral JAK inhibitor, has resulted in clinically meaningful improvements in patient-reported outcomes (PROs), including in key components of pain, fatigue, and physical functioning [13–15]. Improvements in PROs have been observed with UPA as monotherapy [16] and in combination with MTX [13, 15]. Improvements were equivalent to or greater than with anti-TNF inhibitor adalimumab [14]. In patients with inadequate response or intolerance to MTX, UPA treatment significantly improved patient-reported pain and physical functioning [13, 14]. ABA is commonly prescribed as a second-line bDMARD for the treatment of RA, and studies have shown that ABA treatment improves PROs, yet comparisons of ABA to JAK inhibitors are limited [17–22]. Further research is needed to guide treatment decisions, particularly from the patients’ perspective. This post hoc analysis evaluated the impact and benefits of treatment with oral UPA versus intravenous (IV) ABA on PROs at weeks 12 and 24 in a head-to-head comparison in patients with active RA and bDMARD-IR in SELECT-CHOICE [23].

## Materials and methods

### Study design and participants

Full details of the study design of SELECT-CHOICE (NCT03086343) were previously reported [23]. This study was a phase 3, double-blind, randomized clinical trial in patients with active bDMARD-IR RA currently receiving background csDMARD therapy. Patients ≥18 years of age with moderately to severely active RA for ≥3 months on a stable background of csDMARD therapy (≥3 months prior to study entry) were randomized double-blind to receive either oral UPA (15 mg once daily) with IV placebo or IV ABA and oral placebo. This study was not placebo-controlled in that, despite patients receiving an oral or IV placebo, patients knew that they were receiving an active drug, however, they were unaware of which drug they were receiving. Both IV placebo and ABA treatments were administered on day 1 and at weeks 2, 4, 8, 12, 16, and 20 at doses of 500, 750, or 1000 mg, depending on patient weight (<60 kg, 60–100 kg, or >100 kg, respectively). Concomitant use of ≤2 of the following csDMARDs was permitted: MTX, sulfasalazine, hydroxychloroquine, chloroquine, or leflunomide; combination of MTX and leflunomide was not permitted. Eligible patients had no previous exposure to ABA. Data on the primary and ranked secondary outcomes of this study have been published previously [23]. The protocol was approved by independent ethics committee or institutional review board at all study sites. All participants provided written, informed consent prior to enrollment. The registered clinical trial was conducted in accordance with the ethical principles that have their origin in the current Declaration of Helsinki and is consistent with the International Conference on Harmonization Good Clinical Practice and Good Epidemiology Practices, and all applicable local regulatory requirements. All patient data were de-identified and complied with patient confidentiality requirements.

### Patient-reported outcomes

Several PROs were collected to assess the impact of UPA on the patients’ symptoms and HRQOL. The Patient Global Assessment of Disease Activity (PtGA) by visual analog scale (VAS) assessed overall disease severity (range 0–100 mm), with higher scores indicating greater disease activity [24–26]. Pain was measured with the Patient’s Assessment of Pain by VAS (range 0–100 mm), wherein higher scores denoted greater pain [24, 25]. The Health Assessment Questionnaire Disability Index (HAQ-DI) assessed physical functioning, and higher scores (range 0–3) indicated greater physical impairment [24, 25, 27]. The Functional Assessment of Chronic Illness Therapy-Fatigue (FACIT-F) evaluated fatigue on a scale of 0–52, with higher scores indicating less fatigue [25, 28]. The EQ-5D 5-level (EQ-5D-5L) assessed perceptions of overall health, and higher index scores indicated better health [29]. Morning stiffness was reported as duration in minutes, and stiffness severity on a scale of 0–10, with higher values indicating longer lasting or worse morning stiffness [24, 30]. The Work Productivity and Activity Impairment (WPAI) assessment was also used and consists of 4 domains: absenteeism, presenteeism, overall work impairment, and activity impairment. WPAI domain scores were expressed as impairment percentages (scale 0–100%), with higher values indicating greater impairment [31]. The 36-Item Short Form Health Survey (SF-36), which consists of 8 domains (physical functioning, role physical, bodily pain, general health, vitality, social functioning, role emotional, and mental health), and the composite Physical (PCS) and Mental (MCS) Component Summary scores were also assessed; higher scores on SF-36 (range 0–100) indicated better health and functioning [24, 25, 32, 33].

Table 1 shows the scoring ranges, minimal clinically important difference (MCID) values, and normative values, where available, for each PRO. Pain, PtGA, HAQ-DI, and morning stiffness were assessed at all time points (day 1 and weeks 2, 4, 8, 12, 16, 20, and 24). WPAI was assessed at day 1 and weeks 4, 8, 12, and 24; FACIT-F was assessed at day 1 and weeks 4, 8, 12, 16, and 24; and the SF36 and EQ-5D-5L were assessed at day 1 and weeks 4, 12, and 24.

**Table 1:** Patient-reported outcomes measurements and meaningful values

PRO	Range	MCID	Normative value
PtGA	0–100 mm	≥−10 mm [18, 24, 25]	≤20 [ (40)]
Pain (VAS)	0–100 mm	≥−10 mm [24, 25]	
HAQ-DI	0–3	≥−0.22 units [24, 25]	≤0.25 unit [41]
Morning stiffness			
Duration	Duration in minutes	½ baseline mean STD	
Severity	0–10	≥−1 point	
SF-36			
SF–36 PF	0–100	≥5.0 points [24]	77.25^a^
SF–36 RP	0–100		78.19^a^
SF–36 BP	0–100		67.60^a^
SF–36 GH	0–100		69.08^a^
SF–36 VT	0–100		57.11^a^
SF–36 SF	0–100		83.37^a^
SF–36 RE	0–100		86.07^a^
SF–36 MH	0–100		75.61^a^
PCS/MCS	0–100	≥2.5 points [24, 25]	≥50 points [24, 25]
FACIT-F	0–52	≥4.0 points [25]	≥43.6 points [42]
EQ-5D-5L	Index score^b^	≥0.05 points [43]	≥0.92 points [44]
WPAI	0 – 100%	≥7% reduction [18]	

^a^Normative values for SF-36 domains are based on an aged and gender matched population. ^b^The EQ-5D-5L is an index score wherein each section is rated from 1 to 5 and then scores indexed to a value ≤1. A score of 1 would indicate best health state. *BP* bodily pain, *EQ-5D-5L* EQ-5D 5-Level, *FACIT-F* Functional Assessment of Chronic Illness Therapy-Fatigue, *GH* general health, *HAQ-DI* Health Assessment Questionnaire Disability Index, *MCID* minimal clinically important difference, *MCS* Mental Component Summary, *MH* mental health, *PCS* Physical Component Summary, *PF* physical functioning, *PtGA* Patient Global Assessment of Disease Activity, *RE* role emotional, *RP* role physical, *SF* social functioning, *SF-36* 36-Item Short Form Health Survey, *VAS* visual analog scale, *VT* vitality, *WPAI* Work Productivity and Activity Impairment

### Statistical analysis of data

Least squares mean (LSM) changes from baseline to weeks 12 and 24 were assessed based on an analysis of covariance model and comparisons between treatment arms used chi-square tests with significance at the 5% level. The proportion of patients reporting improvements ≥ MCID from baseline through weeks 12 and 24, and those achieving normative values were calculated for UPA and ABA treatment. For each PRO, response rates were only calculated for patients who had non-missing baseline PRO scores and missing values were imputed as non-responses. The incremental numbers needed to treat (NNTs) to demonstrate MCID were calculated as the reciprocal of the response rate differences between UPA and ABA for each PRO at weeks 12 and 24. WPAI work impairment was only calculated for patients who were employed. Time to response, defined as improvement ≥ MCID, was assessed for pain (VAS), HAQ-DI, and duration and severity of morning stiffness and was assessed by Kaplan-Meier analysis and compared using the log-rank test. The PRO endpoints presented in this manuscript were not ranked secondary endpoints and, thus, were not controlled for multiplicity. As such, nominal *P* values are provided throughout.

## Results

### Patient demographics

The study enrolled and randomized 612 patients (UPA, *n*=303; ABA, *n*=309) with a mean age of 56 years and an average RA duration of 12 years (Table 2). Baseline characteristics among the treatment arms were comparable, with nearly one-third of patients enrolled having received 2 or more prior bDMARDs (32%), over 60% having received at least one TNF inhibitor, and over 80% of patients receiving MTX, with or without another csDMARD, at baseline. Over half of the patients enrolled were on an oral steroid at baseline. Baseline PROs were similar between the two treatment groups (Table 3) and reflect the impact of RA on the HRQOL in patients with a long disease duration.

**Table 2:** Patient demographics and RA treatment history

Characteristics	UPA 15 mg (*n*=303)	ABA IV^a^ (*n*=309)
**Age**, mean ± SD	55.3 ± 11.4	55.8 ± 11.9
**Female**, *n* (%)	249 (82)	253 (82)
**White**, *n* (%)	288 (95)	285 (92)
**Duration of RA diagnosis**, mean ± SD	12.4 ± 9.5	11.8 ± 8.3
**Number of prior bDMARDs received**, *n* (%)		
1	206 (68)	202 (65)
2	64 (21)	70 (23)
≥3	29 (10)	35 (11)
**Number of prior TNF inhibitors received**, *n* (%)		
0	40 (13)	36 (12)
1	212 (70)	198 (64)
2	38 (13)	64 (21)
3	13 (4)	10 (3)
≥4	0	1 (0.3)
**Concomitant csDMARDs at baseline**, *n* (%)		
MTX alone	223 (74)	215 (70)
MTX and other csDMARD	30 (10)	38 (12)
csDMARD other than MTX	45 (15)	56 (18)
**Oral steroid at baseline**, *n* (%)	169 (56)	158 (51)

^a^ABA IV at day 1 and weeks 2, 4, 8, 12, 16, and 20 (<60 kg: 500 mg; 60–100 kg: 750 mg; >100 kg: 1000 mg). *ABA* abatacept, *bDMARD* biologic disease-modifying antirheumatic drug, *csDMARD* conventional synthetic disease-modifying antirheumatic drug, *IV* intravenous, *MTX* methotrexate, *RA* rheumatoid arthritis, *SD* standard deviation, *TNF* tumor necrosis factor, *UPA* upadacitinib

**Table 3:** LSM at baseline and change at weeks 12 and 24

PRO assessment	UPA 15 mg (*n*=303)			ABA IV^a^ (*n*=309)		
	Baseline Mean ± SD	Difference at week 12 LSM (95%CI)	Difference at week 24 LSM (95%CI)	Baseline Mean ± SD	Difference at week 12 LSM (95%CI)	Difference at week 24 LSM (95%CI)
PtGA	66.8 ± 19.9	− 33.9* (− 37.1, − 30.6)	− 38.7 (− 42.1, − 35.3)	69.6 ± 20.8	− 28.4 (− 31.5, − 25.2)	− 36.9 (− 40.2, − 33.5)
Pain (VAS)	68.0 ± 20.2	− 35.3* (− 38.5, − 32.0)	− 41.5 (− 44.8, − 38.2)	70.8 ± 19.5	− 30.0 (− 33.2, − 26.8)	− 37.7 (− 41.0, − 34.4)
HAQ-DI	1.7 ± 0.6	− 0.65* (− 0.72, − 0.57)	− 0.79* (− 0.87, − 0.70)	1.7 ± 0.6	− 0.48 (− 0.55, − 0.40)	− 0.66 (− 0.74, − 0.58)
FACIT-F	25.3 ± 11.2	9.6 (8.3, 10.9)	10.7 (9.4, 12.1)	25.6 ± 11.3	8.4 (7.1, 9.6)	10.3 (9.0, 11.7)
Morning stiffness						
Severity^b^	6.4 ± 2.3	− 3.3* (− 3.6, − 3.0)	− 3.9* (− 4.2, − 3.6)	6.5 ± 2.4	− 2.8 (− 3.1, − 2.5)	− 3.5 (− 3.8, − 3.2)
Duration^c^	185.5 ± 280.5	− 74.4 (− 110.1, − 38.6)	− 97.1 (− 134.1, − 60.1)	207.2 ± 311.5	− 70.8 (− 105.9, − 35.8)	− 92.8 (− 129.5, − 56.0)
EQ-5D-5L index score	0.5 ± 0.3	0.26* (0.23, 0.28)	0.28 (0.26, 0.30)	0.5 ± 0.3	0.21 (0.19, 0.24)	0.25 (0.23, 0.28)
WPAI domains						
Absenteeism	22.4 ± 30.8	− 11.0 (− 16.5, − 5.4)	− 15.8 (− 21.2, − 10.4)	21.6 ± 30.7	− 13.0 (− 18.8, − 7.1)	− 10.5 (− 16.3, − 4.6)
Presenteeism	54.3 ± 23.3	− 26.0* (− 32.4, − 19.6)	− 26.0 (− 32.6, − 19.4)	52.7 ± 27.3	− 17.5 (− 24.2, − 10.8)	− 23.0 (− 30.3, − 15.7)
Overall work impairment	62.2 ± 26.8	− 25.7 (− 32.8, − 18.5)	− 29.8 (− 37.0, − 22.5)	60.9 ± 30.0	− 22.1 (− 29.7, − 14.6)	− 25.5 (− 33.4, − 17.7)
Activity impairment	64.4 ± 22.0	− 26.8* (− 30.1, − 23.5)	− 31.5* (− 35.0, − 28.0)	67.2 ± 22.5	− 21.0 (− 24.2, − 17.7)	− 25.8 (− 29.3, − 22.3)
SF-36 composite scores						
SF-36 PCS	31.2 ± 7.4	9.6* (8.6, 10.7)	11.0* (9.8, 12.1)	31.3 ± 6.8	7.0 (6.0, 8.1)	9.4 (8.3, 10.5)
SF-36 MCS	43.7 ± 11.6	5.6 (4.5, 6.8)	6.4 (5.2, 7.7)	42.8 ± 12.1	5.8 (4.7, 7.0)	6.4 (5.2, 7.6)
SF-36 domain scores						
SF-36 PF	30.7 ± 8.4	8.6* (7.5, 9.8)	10.1 (8.9, 11.3)	30.5 ± 8.3	5.9 (4.8, 7.1)	8.7 (7.5, 9.9)
SF-36 RP	33.4 ± 7.6	7.9 (6.9, 9.0)	9.6 (8.4, 10.7)	32.8 ± 7.7	7.0 (5.9, 7.9)	8.5 (7.4, 9.7)
SF-36 BP	32.9 ± 6.5	12.2* (11.1, 13.4)	13.4* (12.3, 14.6)	32.5 ± 6.6	9.8 (8.7, 10.9)	11.7 (10.6, 12.9)
SF-36 GH	37.3 ± 8.9	6.7* (5.5, 7.9)	7.1 (6.0, 8.3)	37.3 ± 7.9	5.0 (3.8, 6.1)	6.4 (5.2, 7.5)
SF-36 VT	40.2 ± 9.6	8.6 (7.4, 9.9)	10.0 (8.6, 11.3)	40.3 ± 9.3	7.7 (6.4, 8.9)	9.3 (8.0, 10.7)
SF-36 SF	37.7 ± 10.5	7.8 (6.6, 8.9)	9.3 (8.1, 10.5)	37.4 ± 10.6	6.6 (5.5, 7.8)	8.0 (6.7, 9.2)
SF-36 RE	39.2 ± 11.3	5.5 (4.4, 6.7)	6.8 (5.6, 8.1)	37.7 ± 11.9	5.5 (4.3, 6.7)	6.5 (5.3, 7.8)
SF-36 MH	41.0 ± 11.1	6.9 (5.7, 8.1)	7.3 (6.1, 8.6)	40.2 ± 11.4	6.3 (5.1, 7.5)	7.1 (5.9, 8.4)

^a^ABA IV at day 1 and weeks 2, 4, 8, 12, 16, and 20 (<60 kg: 500 mg; 60–100 kg: 750 mg; >100 kg: 1000 mg). ^b^Assessed on a numeric scale of 1–10, with 10 being the worst level. ^c^Duration in minutes. *ABA* abatacept, *AM* morning, *BP* bodily pain, *EQ-5D-5L* (index score) EQ-5D 5-Level, *FACIT-F* Functional Assessment of Chronic Illness Therapy-Fatigue, *GH* general health, *HAQ-DI* Health Assessment Questionnaire Disability Index, *IV* intravenous, *LSM* least squares mean, *MCS* Mental Component Summary, *MH* mental health, *PCS* Physical Component Summary, *PF* physical functioning, *PRO* patient-reported outcome, *PtGA* Patient Global Assessment of Disease Activity, *QD* once daily, *RE* role emotional, *RP* role physical, *SF* social functioning, *SF-36* 36-Item Short Form Health Survey, *UPA* upadacitinib, *VAS* visual analog scale, *VT* vitality, *WPAI* Work Productivity and Activity Impairment. *P<0.05 for UPA vs ABA. *P* values represent statistical significance between groups

### LSM changes from baseline

At week 12, UPA treatment resulted in statistically significant improvements in PtGA, pain, HAQ-DI, severity of AM stiffness, EQ-5D-5L, WPAI activity impairment and presenteeism domains, three SF-36 domains (physical functioning, bodily pain, and general health), and the SF-36 PCS score (*p*<0.05, Table 3) as compared to improvements with ABA. At week 24, changes from baseline were maintained in UPA-treated patients and a significant difference persisted between UPA- and ABA-treated patients in HAQ-DI, severity of AM stiffness, WPAI activity impairment domain, and the SF-36 PCS and bodily pain domain scores; changes from baseline were similar between groups for the remaining PROs.

### Proportion of patients reporting improvements ≥ MCID in PROs at weeks 12 and 24

**Fig. 1:**
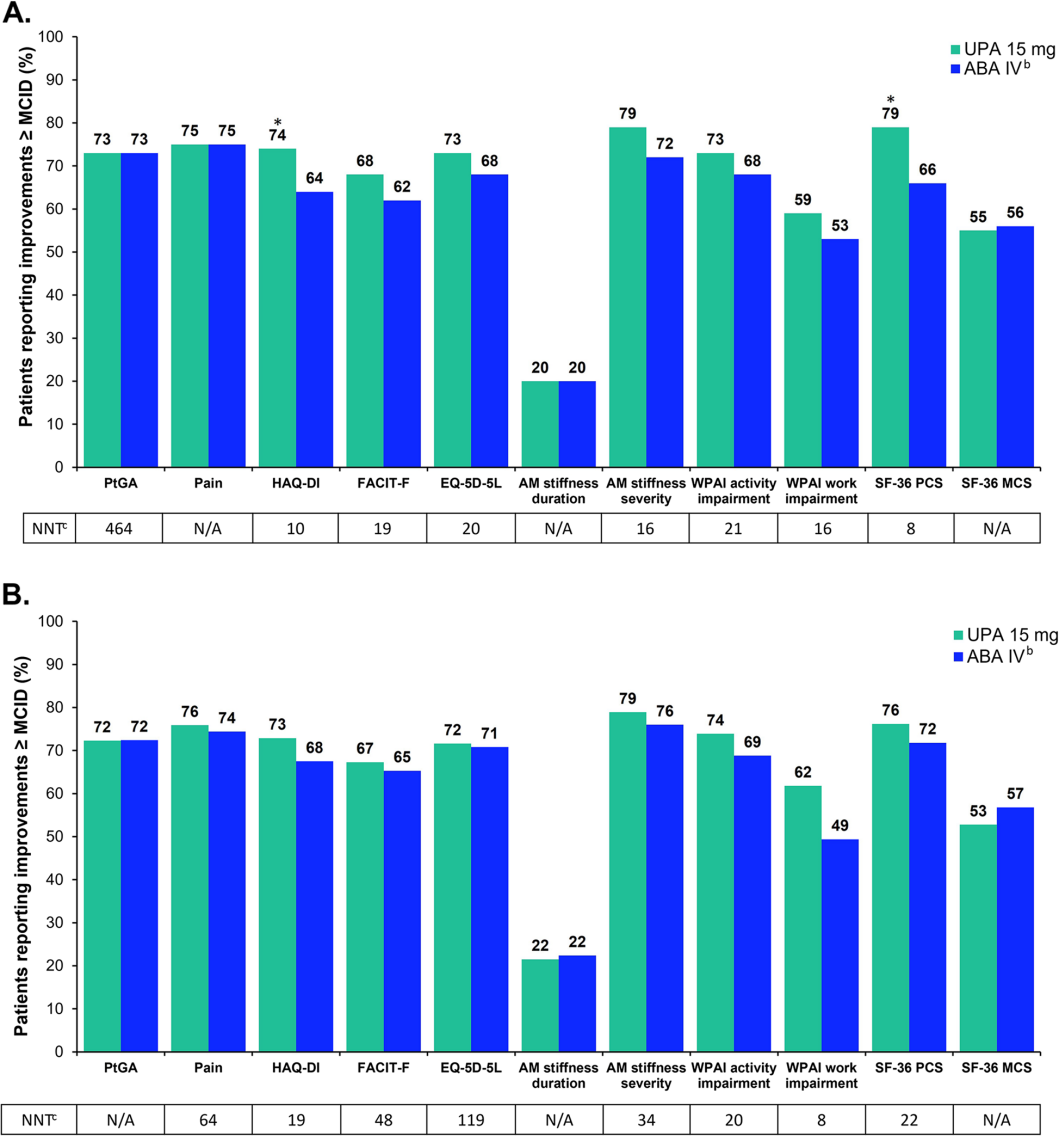
Proportion of patients reporting improvements ≥ MCID^a^ in PROs at weeks 12 (**A**) and 24 (**B**). ^a^MCID was defined as reduction of ≥10 mm for PtGA and pain, ≥1 for severity of AM stiffness, reduction of ≥0.22 units for HAQ-DI, increase of ≥4 points for FACIT-F, proxied at one-half standard deviation for duration of AM stiffness, increase of ≥0.05 points for EQ-5D-5L, reduction of 7% in score for WPAI, and increase of ≥2.5 points for SF-36 PCS and MCS. ^b^ABA IV at day 1 and weeks 2, 4, 8, 12, 16, and 20 (<60 kg: 500 mg; 60–100 kg: 750 mg; >100 kg: 1,000 mg). ^c^NNTs are for UPA vs ABA. *ABA *abatacept, *AM* morning, *EQ-5D-5L* *(index score)*, EQ-5D 5-Level, *FACIT-F* Functional Assessment of Chronic Illness Therapy-Fatigue, *HAQ-DI* Health Assessment Questionnaire Disability Index, *IV* intravenous, *MCID* minimal clinically important difference, *MCS* Mental Component Summary. *NNT *number needed to treat, *PCS* Physical Component Summary, *PRO* patient-reported outcome, *PtGA* Patient Global Assessment of Disease Activity, *SF-36* 36-Item Short Form Health Survey, *UPA* upadacitinib, *VAS* visual analog scale, *WPAI *Work Productivity and Activity Impairment. *P<0.05 for UPA vs ABA. *P* values represent statistical significance between treatment groups

**Fig. 2:**
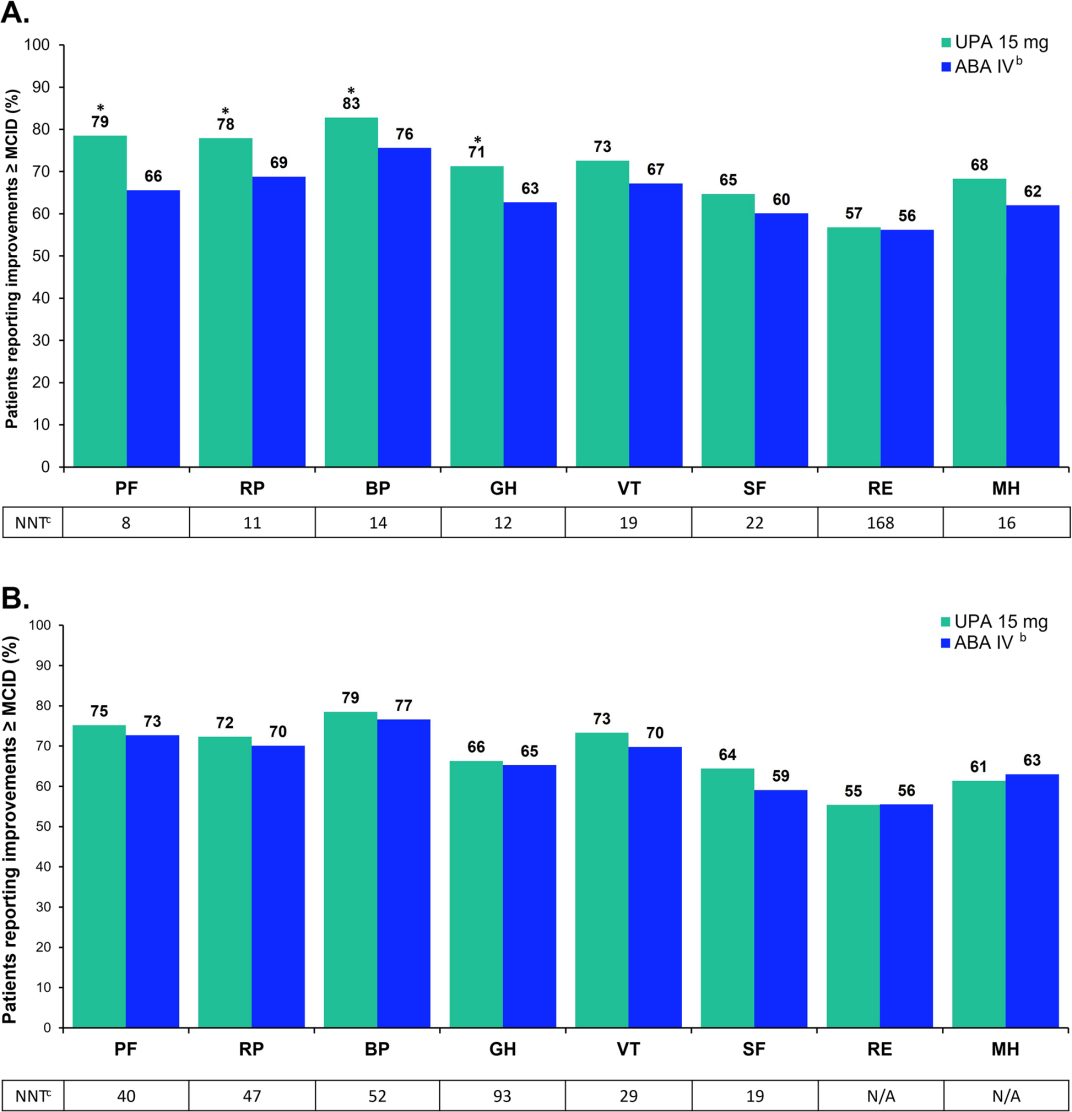
Proportion of patients reporting improvements ≥ MCID^a^ in SF-36 at weeks 12 (**A**) and 24 (**B**). ^a^MCID was defined as increase ≥5.0 for all SF-36 domains. ^b^ABA IV at day 1 and weeks 2, 4, 8, 12, 16, and 20 (<60 kg: 500 mg; 60–100 kg: 750 mg; >100 kg: 1000 mg). ^c^NNTs are for UPA vs ABA. *ABA* abatacept, *BP *bodily pain, *GH* general health, *IV* intravenous, *MCID* minimal clinically important difference, *MH* mental health, *PF* physical functioning, *RE* role emotional, *RP* role physical, *SF* social functioning, *SF-36* 36-Item Short Form Health Survey, *UPA* upadacitinib, *VT* vitality. *P<0.05 for UPA vs ABA. *P* values represent statistical significance between treatment groups

Compared with ABA at week 12, significantly more UPA-treated patients reported improvements ≥ MCID in HAQ-DI. Similar proportions of patients reported clinically meaningful improvements in the ability to perform work and daily activities as demonstrated by WPAI scores. Likewise, similar proportions of patients reported improvements ≥ MCID across other PROs (Fig. 1A). Among the SF-36 domain scores, a significantly greater proportion of patients receiving UPA, as compared with ABA, reported improvements ≥ MCID on physical functioning, role physical, bodily pain, and general health (Fig. 2A). Likewise, clinically meaningful improvements in SF-36 PCS scores were reported in significantly more UPA-treated patients (Fig. 1A). Improvements in the other 4 domains were similar between groups. At week 24, more UPA-treated patients reported improvements ≥ MCID in most PROs compared with ABA-treated patients; however, these differences were not statistically significant (Figs. 1Band 2B).

### Proportion of patients achieving normative values in PROs at baseline and weeks 12 and 24

At baseline, few patients reported having normative PRO scores (i.e., values consistent with those reported by patients without disease; Fig. S1 and S2). Not all PROs assessed in this study have known normative values, thus achievement of normative values is only reported for a subset of PROs. The percentage of patients reporting normative values at baseline, for both UPA and ABA groups, ranged from 1% (SF-36 PCS) to 29% (SF-36 MCS). By week 12, the percentages of UPA- vs ABA-treated patients achieving normative values were significantly greater in PtGA (37% vs 23%), HAQ-DI (18% vs 10%), and EQ-5D-5L (22% vs 13%, Fig. S1). Likewise, a significantly greater proportion of patients receiving UPA reported normative values for SF-36 PCS (17% vs 8%, Fig. S1), physical functioning (21% vs 11%), bodily pain (33% vs 23%), and general health (24% vs 17%) domains (Fig. S2). At week 24, significantly more UPA- vs ABA-treated patients achieved normative PRO scores in PtGA (44% vs 34%), HAQ-DI (23% vs 16%), and SF-36 PCS (21% vs 12%) and role physical (22% vs 16%) and bodily pain (38% vs 29%) domains (*p*<0.05; Figs. S1 and S2). While more UPA-treated patients achieved normative values in the remaining PROs at week 24 compared to ABA-treated patients, the differences between groups were not statistically significant.

### Time to treatment response

The time to response (≥MCID), as measured by HAQ-DI, was significantly shorter for UPA- vs ABA-treated patients (medians: 2 weeks vs 4 weeks, *P*<0.01 [data not shown]). The median time to response was not statistically significantly different for UPA- versus ABA-treated patients in pain (2 weeks vs 4 weeks). There was no difference in the median time to response for morning stiffness severity or morning stiffness duration.

## Discussion

JAK inhibitors, including UPA, are a newer class of treatment for RA in comparison to other biologics such as ABA, which have been commonly accepted therapies for patients with RA over the past 20 years. Understanding the efficacy of newer therapies, especially as they compare to more established ones, is important because nearly half of patients do not adequately respond to first-line csDMARDs and up to 66% do not adequately respond to second-line biologics [7, 8]. Thus, these patients represent a population that may be more difficult to treat. The SELECT-CHOICE study is a phase 3 trial that is a direct head-to-head comparison of efficacy, safety, and PROs between UPA and ABA in a bDMARD-IR population [23]. Primary efficacy data demonstrated that UPA was superior to ABA in the change from baseline in DAS28-CRP components and achievement of remission after 12 weeks of treatment; after 24 weeks of treatment, change from baseline in DAS28-CRP components remained numerically greater in UPA- vs ABA-treated patients but were not statistically significant [23]. As a supplement to the clinical efficacy results, analysis of other secondary endpoints showed that both UPA and ABA demonstrated improvement in PROs; however, UPA treatment resulted in more significant and clinically meaningful improvements in PROs at 12 weeks when compared with ABA. Early differences between the treatments were seen in key domains of physical functioning, pain, and general health, with improvements in HAQ-DI observed 2 weeks earlier in UPA- vs ABA-treated patients. In this study, patients treated with UPA achieved significantly greater improvements from baseline in PtGA, pain, HAQ-DI, and FACIT-F as compared with ABA at week 12. Likewise, SF-36 PCS, 3 SF-36 domains (physical functioning, bodily pain, and general health), and 2 WPAI domains (presenteeism and activity impairment) showed significant improvement with UPA vs ABA; at week 12 similar improvements were noted for UPA and ABA in other PROs. At week 24, the change from baseline in HAQ-DI, severity of AM stiffness, WPAI activity impairment domain, and SF-36 PCS and bodily pain domain scores in UPA-treated patients remained statistically significant compared with ABA-treated patients; changes from baseline were similar between groups for the remaining PROs. This study also demonstrated that more patients receiving UPA achieved normative values in PtGA, HAQ-DI, SF-36 PCS, and SF-36 bodily pain domain at both weeks 12 and 24 as compared with ABA-treated patients; UPA-treated patients also had significantly better improvement in the SF-36 role physical domain at week 24 compared with ABA-treated patients. Despite no statistically significant differences in the proportion of patients achieving MCID in these PROs at week 24, these data would suggest that improvements in these PROs with UPA treatment may be more substantial than those improvements observed with ABA treatment based on normative value achievements. The improvements in PROs reported with UPA in this study are similar to those improvements seen with UPA previously in csDMARD-IR and bDMARD-IR patient populations [13–15, 23]. Data from SELECT-COMPARE, which compared UPA, placebo, and adalimumab treatment with a background of MTX at 12 weeks also demonstrated significant improvements with UPA in PtGA, pain, HAQ-DI, morning stiffness severity, FACIT-F, SF-36 PCS, and 6/8 SF-36 domain scores as compared with adalimumab and placebo [14]. Importantly, SELECT-COMPARE enrolled patients who had inadequate response or intolerance to MTX, whereas this study enrolled bDMARD-IR patients, who represent a difficult-to-treat population with a greater, unmet medical need.

Assessment of PROs in chronic disease is key to understanding patient perspectives and should be included when analyzing study drug efficacy. PROs are useful tools to measure the impact of chronic illness on daily living and work abilities because these also impact healthcare resource utilization and overall economic burden of disease. Likewise, PROs can influence treatment decisions and provide a more customized approach to disease management, especially when treatments are comparable [34]. When selecting treatments, time to response, route of administration, and quantity of doses taken per day are also important factors to consider as they may greatly affect patient’s perception of efficacy and overall treatment adherence [35, 36]. Patients with RA frequently experience pain, fatigue, and impaired physical functioning and these may have negative impacts on their HRQOL [1–4]. Fatigue and pain are also associated with reductions in mental well-being and the ability of patients to perform daily activities and maintain employment [4, 37, 38]. In the current study, improvements in physical functioning (HAQ-DI) and severity of morning stiffness were observed as early as 2 weeks after treatment initiation with UPA. After 12 weeks of treatment, greater proportions of UPA- vs ABA-treated patients reported clinically meaningful improvements in physical functioning and in 4 of 8 SF-36 domain and PCS scores. The proportion of UPA- vs ABA-treated patients reporting achieving normative values after 12 weeks treatment was significantly greater in PtGA, physical functioning, general HRQOL by EQ-5D-5L, and SF-36 PCS and 3 of 8 domain scores (i.e., physical functioning, bodily pain, and general health). The proportion of UPA- vs ABA-treated achieving normative values was significantly greater for PtGA, physical functioning, and SF-36 PCS and bodily pain and role physical domain scores at week 24, with significant proportions of patients also achieving normative values for the SF-36 role physical domain. Similar percentages of patients (over half) treated with UPA or ABA achieved clinically meaningful reductions in work and activity impairment at week 12. At week 24, over 68% of UPA or ABA-treated patients had clinically meaningful improvement in activity impairment; 62% of UPA-treated patients had clinically meaningful improvement in work impairment vs only 49% of ABA-treated patients. Likewise, similar percentages of patients treated with UPA or ABA also achieved clinically meaningful reductions in the key symptom of fatigue. Importantly, patients reported shorter median response times to improvements in physical functioning with UPA treatment compared with ABA. Together, these results suggest that UPA may lead to meaningful early improvements in key PROs that are important to patients, including fatigue, pain, physical functioning, and ability to perform work and daily activities.

There are both strengths and limitations to this study. Strengths of the study include the utilization of several validated PROs that reflect the different aspects of the patient experience. To our knowledge, this is the first clinical study comparing a JAK inhibitor to ABA in a bDMARD-IR population. This study fills the gap by providing important data on patient-perceived efficacy in this population. The use of MCIDs and normative values allow for the data to be clinically meaningful and interpretable for patients and physicians. Blinded and randomized study design allows for unbiased reporting from each patient and mitigates biases due to differences between treatment groups. Limitations of the study include the collection of PROs at fixed visits, sometimes weeks apart, with no day-to-day data available. Prolonged recall of such dynamic symptoms may introduce recall bias that could affect patient perceptions of efficacy [39]. Although patients did receive either IV or oral placebo in combination with active therapy, this trial was not placebo-controlled since patients were aware that they were receiving an active treatment. This may impact on patients’ perception of drug efficacy. The PROs presented here were not multiplicity controlled, ranked secondary endpoints, thus all significance values are nominal. Imputation of missing data as non-response may lead to an underestimation of the true response rate for each PRO. The time frame of this analysis was relatively short (24 weeks), thus additional studies are needed to determine if the patient-reported improvements observed are maintained long-term.

## Conclusions

Treatment with UPA or ABA resulted in rapid and clinically meaningful improvements in PROs among bDMARD-IR patients with moderately to severely active RA. Overall, greater improvements from baseline in PROs with UPA vs ABA treatment, especially in the key domains of physical functioning, pain, and general health, were observed after 12 and 24 weeks of treatment. Although the proportion of patients achieving ≥MCID were similar between UPA- and ABA-treated patients at week 24, the numerically greater improvements from baseline in PROs, coupled with higher percentages of patients achieving normative values, suggest that improvement in those PROs may be more substantial with UPA vs ABA treatment. Moreover, these data suggest that patients receiving UPA had faster therapeutic response times, as seen by earlier meaningful improvement in PROs, compared with ABA-treated patients.

## Supplementary Information

**Additional file 1: Figure S1.** Proportion of Patients Reporting PRO Scores ≥ Normative Values at Baseline and weeks 12 and 24. ^a^ABA IV at day 1 and weeks 2, 4, 8, 12, 16, and 20 (<60 kg: 500 mg; 60–100 kg: 750 mg; >100 kg: 1,000 mg). ABA, abatacept; BL, baseline; EQ-5D-5L (index score), EQ-5D 5-Level; FACIT-F, Functional Assessment of Chronic Illness Therapy-Fatigue; HAQ-DI, Health Assessment Questionnaire Disability Index; IV, intravenous; LDA, low disease activity; MCS, Mental Component Summary; PCS, Physical Component Summary; PRO, patient-reported outcome; PtGA, Patient Global Assessment of Disease Activity; SF-36, 36-Item Short Form Health Survey; UPA, upadacitinib. *P<0.05 for UPA vs ABA. †*P*=0.05 for UPA vs ABA.

**Additional file 2: Figure S2.** Proportion of Patients Reporting SF-36 Scores ≥ Normative Values at Baseline and weeks 12 and 24. ^a^ABA IV at day 1 and weeks 2, 4, 8, 12, 16, and 20 (<60 kg: 500 mg; 60–100 kg: 750 mg; >100 kg: 1,000 mg). ABA, abatacept; BL, baseline; BP, bodily pain; GH, general health; IV, intravenous; MH, mental health; PF, physical functioning; RE, role emotional; RP, role physical; SF, social functioning; SF-36, 36-Item Short Form Health Survey; UPA, upadacitinib; VT, vitality. *P<0.05 for UPA vs ABA.

## Data Availability

AbbVie is committed to responsible data sharing regarding the clinical trials we sponsor. Access is provided to anonymized, patient and trial-level data (analysis data sets), as well as other information (e.g., protocols and Clinical Study Reports) from AbbVie-sponsored Phase II-IV global interventional clinical trials conducted in patients (completed as of May 2004, for products and indications approved in either the USA or the European Union), as long as the trials are not part of an ongoing or planned regulatory submission). This includes requests for clinical trial data for unlicensed products and indications. Access to this clinical trial data can be requested by any qualified researchers who engage in rigorous, independent scientific research, and will be provided following review and approval of a research proposal and Statistical Analysis Plan (SAP) and execution of a Data Sharing Agreement (DSA). Data requests can be submitted at any time and the data will be accessible for 12 months, with possible extensions considered. For more information on the process, or to submit a request, visit the following link: https://www.abbvie.com/our-science/clinical-trials/clinical-trials-data-and-informationsharing/data-and-information-sharing-with-qualified-researchers.html.

## Abbreviations

ABA: Abatacept
bDMARD: Biologic disease-modifying antirheumatic drugs
bDMARD-IR: Biologic disease-modifying antirheumatic drugs inadequate response or intolerant
BL: Baseline
CRP: C-reactive protein
csDMARD: Conventional synthetic disease-modifying antirheumatic drugs
DAS: Disease activity score
DAS28: Disease activity score for 28 joints
EQ-5D-5L: European Quality of Life 5-Dimension 5-Level
FACIT-F: Functional Assessment of Chronic Illness Therapy - Fatigue
HAQ-DI: Health Assessment Questionnaire Disability Index
HRQOL: Health-related quality of life
IV: Intravenous
JAK: Janus kinase
LSM: Least square mean
MCID: Minimal clinically important difference
MCS: Mental Component Summary
MTX: Methotrexate
PCS: Physical Component Summary
PRO: Patient-reported outcome
PtGA: Patient Global Assessment of Disease Activity
QOL: Quality of life
RA: Rheumatoid arthritis
SF-36: 36-Item Short Form Health Survey
UPA: Upadacitinib
VAS: Visual analog scale
WPAI: Work Productivity and Activity Impairment
