# Correction: Patient-reported outcomes of upadacitinib versus abatacept in patients with rheumatoid arthritis and an inadequate response to biologic disease-modifying antirheumatic drugs: 12- and 24-week results of a phase 3 trial

**DOI:** 10.1186/s13075-022-02940-5

**Published:** 2022-11-03

**Authors:** Martin Bergman, Namita Tundia, Naomi Martin, Jessica L. Suboticki, Jayeshkumar Patel, Debbie Goldschmidt, Yan Song, Grace C. Wright

**Affiliations:** 1 Drexel University College of Medicine, Philadelphia, PA USA; 2 AbbVie Inc, North Chicago, IL USA; 3 Analysis Group, Inc, Boston, MA USA; 4 Grace C Wright MD PC; Association of Women in Rheumatology; United Rheumatology, New York, NY USA

**Correction: Arthritis Res Ther 24, 155 (2022)**

**https://doi.org/10.1186/s13075-022-02813-x**

Following publication of the original article [1], the authors reported an error in Additional files 1 and 2 as the axes on the supplemental figures were unclear. The additional files were updated.

The original article [1] has been updated.

## Supplementary Information

**Additional file 1: Figure S1.** Proportion of Patients Reporting PRO Scores ≥ Normative Values at Baseline and weeks 12 and 24. ^a^ABA IV at day 1 and weeks 2, 4, 8, 12, 16, and 20 (<60 kg: 500 mg; 60–100 kg: 750 mg; >100 kg: 1,000 mg). ABA, abatacept; BL, baseline; EQ-5D-5L (index score), EQ-5D 5-Level; FACIT-F, Functional Assessment of Chronic Illness Therapy-Fatigue; HAQ-DI, Health Assessment Questionnaire Disability Index; IV, intravenous; LDA, low disease activity; MCS, Mental Component Summary; PCS, Physical Component Summary; PRO, patient-reported outcome; PtGA, Patient Global Assessment of Disease Activity; SF-36, 36-Item Short Form Health Survey; UPA, upadacitinib. *P<0.05 for UPA vs ABA. †*P*=0.05 for UPA vs ABA.

**Additional file 2: Figure S2.** Proportion of Patients Reporting SF-36 Scores ≥ Normative Values at Baseline and weeks 12 and 24. ^a^ABA IV at day 1 and weeks 2, 4, 8, 12, 16, and 20 (<60 kg: 500 mg; 60–100 kg: 750 mg; >100 kg: 1,000 mg). ABA, abatacept; BL, baseline; BP, bodily pain; GH, general health; IV, intravenous; MH, mental health; PF, physical functioning; RE, role emotional; RP, role physical; SF, social functioning; SF-36, 36-Item Short Form Health Survey; UPA, upadacitinib; VT, vitality. *P<0.05 for UPA vs ABA.
