# Supplementary figures and images for: Patient-reported outcomes of upadacitinib versus abatacept in patients with rheumatoid arthritis and an inadequate response to biologic disease-modifying antirheumatic drugs: 12- and 24-week results of a phase 3 trial
Source: Arthritis Res Ther. 2022 Jun 24;24:155. doi: 10.1186/s13075-022-02813-x (PMC9229430; doi:10.1186/s13075-022-02813-x)

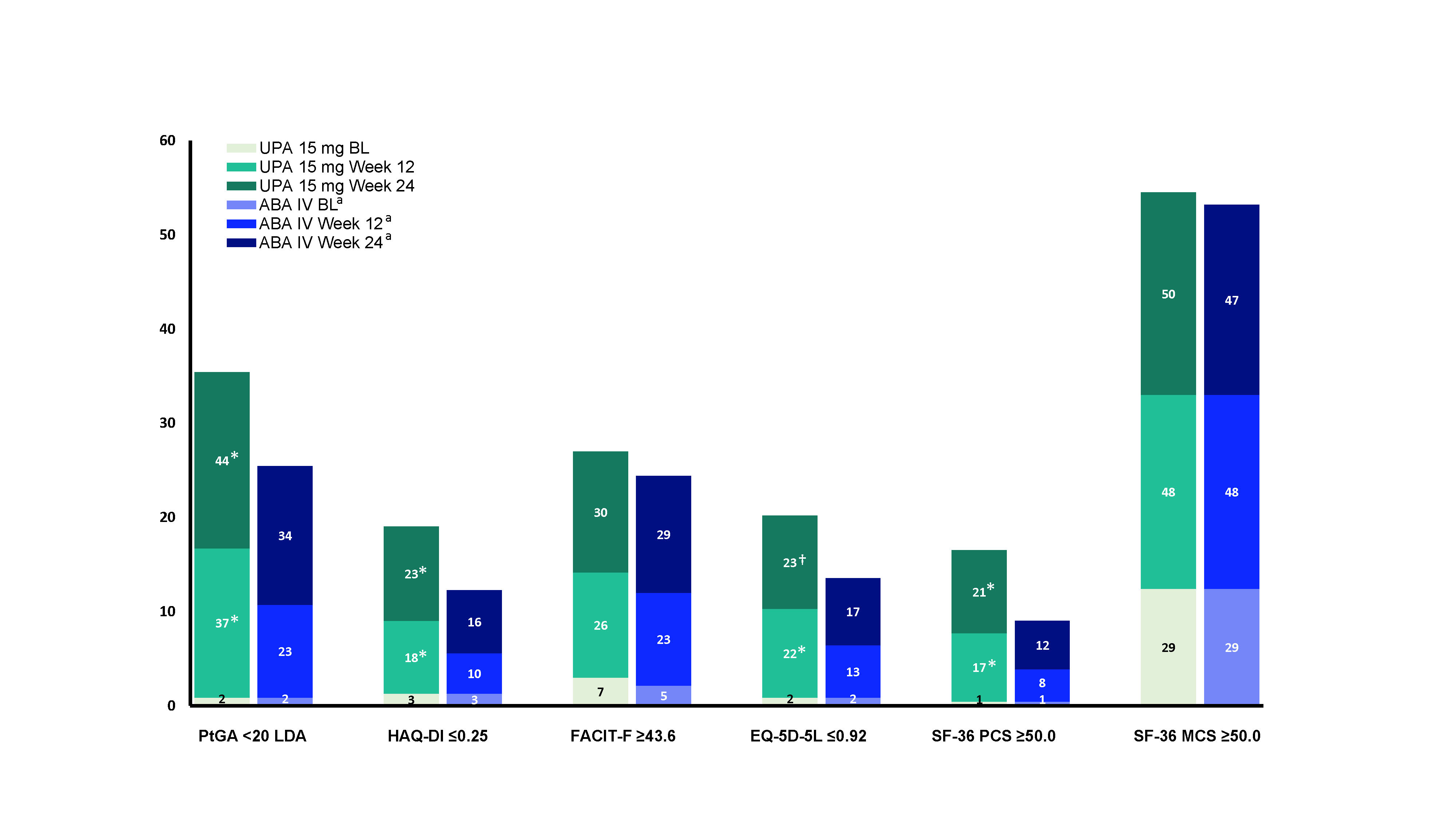

Supplement: Supplementary file 1 — Additional file 1: Figure S1. Proportion of Patients Reporting PRO Scores ≥ Normative Values at Baseline and weeks 12 and 24. aABA IV at day 1 and weeks 2, 4, 8, 12, 16, and 20 (<60 kg: 500 mg; 60–100 kg: 750 mg; >100 kg: 1,000 mg). ABA, abatacept; BL, baseline; EQ-5D-5L (index score), EQ-5D 5-Level; FACIT-F, Functional Assessment of Chronic Illness Therapy-Fatigue; HAQ-DI, Health Assessment Questionnaire Disability Index; IV, intravenous; LDA, low disease activity; MCS, Mental Component Summary; PCS, Physical Component Summary; PRO, patient-reported outcome; PtGA, Patient Global Assessment of Disease Activity; SF-36, 36-Item Short Form Health Survey; UPA, upadacitinib. *P<0.05 for UPA vs ABA. †P=0.05 for UPA vs ABA. [file 13075_2022_2813_MOESM1_ESM.jpg]

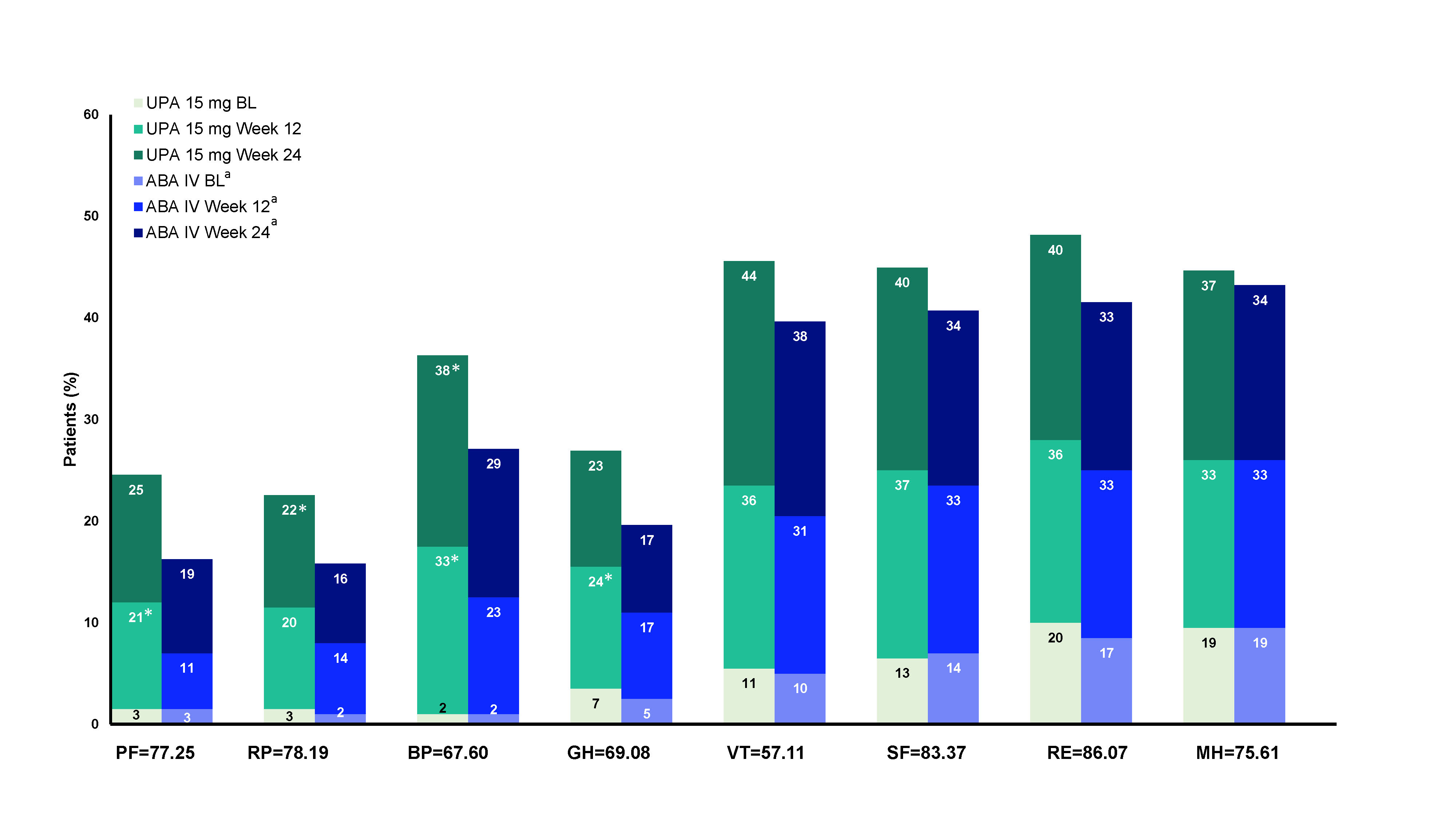

Supplement: Supplementary file 2 — Additional file 2: Figure S2. Proportion of Patients Reporting SF-36 Scores ≥ Normative Values at Baseline and weeks 12 and 24. aABA IV at day 1 and weeks 2, 4, 8, 12, 16, and 20 (<60 kg: 500 mg; 60–100 kg: 750 mg; >100 kg: 1,000 mg). ABA, abatacept; BL, baseline; BP, bodily pain; GH, general health; IV, intravenous; MH, mental health; PF, physical functioning; RE, role emotional; RP, role physical; SF, social functioning; SF-36, 36-Item Short Form Health Survey; UPA, upadacitinib; VT, vitality. *P<0.05 for UPA vs ABA. [file 13075_2022_2813_MOESM2_ESM.jpg]
